# Sympatric pikas partition microhabitats differently across a spatial-elevational gradient

**DOI:** 10.1098/rsos.250865

**Published:** 2026-10-14

**Authors:** Harsha K. Kumar, Dhanesh Ponnu, Senan D'Souza, Dhiraj Das, Skarma Thundup, Nandini Rajamani

**Affiliations:** ^1^ IISER Tirupati, Tirupati, India; ^2^ Tata Institute of Fundamental Research Hyderabad, Hyderabad, Telangana, India; ^3^ Ashoka Trust for Research in Ecology and the Environment, Bengaluru, Karnataka 560064, India; ^4^ Wildlife Research and Conservation Society, Pune, Maharashtra, India

**Keywords:** niche, competition, spatial segregation, microhabitat selection, sympatry, Ladakh, Ochotonidae

## Abstract

How closely related, ecologically similar species co-occur and navigate competition remains a central question in community ecology. Microhabitat partitioning in patchy environments can support the co-existence of sympatric species on small spatial scales. We quantified niche spaces and directional niche overlaps of three cold-adapted, high-altitude pikas (*Ochotona* spp.) from the Trans-Himalayas to understand their responses to interspecific competition across elevational and spatial scales. Vegetation- and landscape-level features were documented across elevational bands. The social, burrowing *O. nubrica* preferred tall stands and high shrub cover on pastures (2800–4500 m). Conversely, the social, burrowing *O. ladacensis* emerged as a high-elevation specialist (4200–6000 m), occupying stunted alpine scrub and steppes. The asocial, rock-dwelling *O. macrotis* preferred microhabitats with high rock cover across elevations (2800–6000 m), associating with significantly greener microhabitats at lower elevations in summer. Visualizing directional niche overlaps revealed scale-dependent patterns of niche segregation; species functioning as generalists at large spatial scales behaved as specialists at smaller scales. As climate change and shifting land-use patterns threaten cold-adapted Himalayan specialists, this nuanced understanding of microhabitat associations in sympatric zones is vital for informing conservation planning.

## 1. Introduction

Interspecific competition is one of the most potent forces that structure ecological communities. The theoretical concept defining the conditions for species co-existence is the Hutchinsonian niche (an *n*-dimensional hypervolume), which defines the full range of biotic and abiotic factors that an animal can survive and reproduce in [1,2]. Within this niche space, species are often placed along a generalist–specialist continuum [3–8]. This categorization presents problems as it is typically based on multiple metrics (the capacity of species to occupy multiple habitats, resource utilization width, dispersal abilities and commensality) [9,10]. For instance, species could be habitat specialists but resource generalists [11,12] or urban specialists but resource generalists [13–15]. To truly understand co-existence patterns in nature, we must move beyond these static descriptions and quantify how species’ realized niches respond to ecological competition [3–8] at different spatial scales [16,17].

Community assembly is primarily governed by abiotic and biotic filtering, where physiological limits and biological interactions limit species occurrence and co-existence [18,19]. Interspecific competition is known to alter the niche width of species [20–23]. Differential habitat/microhabitat use in heterogeneous/patchy environments allows specialists and generalists of closely related species to co-exist by alleviating competition through ecological specialization at both spatial [24–27] and temporal scales [28,29]. Although microhabitat partitioning can reduce competition [29–31], it is often difficult to establish whether competition has driven this choice over and above other biotic and abiotic filtering mechanisms [32–34]. Thus, quantifying the precise relationships between microhabitat partitioning and scale/temporal dynamics of species interactions is crucial for understanding the maintenance of community diversity [35–37]. However, such scale-dependent patterns of interactions and how they affect the niches of species have only been explored in a handful of studies [16,17]. Quantifying the resulting shifts and reductions in realized niche space is a critical ecological signature of competitive outcomes, as definitive proof often requires measuring experimental population growth, which is affected by a whole range of other biotic factors, including predation and disease [21,22,38]. This study advances existing work by quantifying scale-dependent niche shifts in a community of high-altitude, cold-adapted mammals across a dramatic elevational gradient. Crucially, our system allowed us to test the interaction between phylogenetic history and strong functional divergence (rock-dwelling versus burrowing lifestyle) as drivers of niche space utilization.

Pikas are high-elevation, cold-adapted small mammals that face significant threats of extirpation due to climate change [39–41]. The Trans-Himalayas of Ladakh, India (see figure 1), offer a unique system to study co-existence, as they host three pika species that occur in varying degrees of sympatry across a large elevational and spatial gradient. These three species occupy distinct microhabitats.

**Figure 1.**
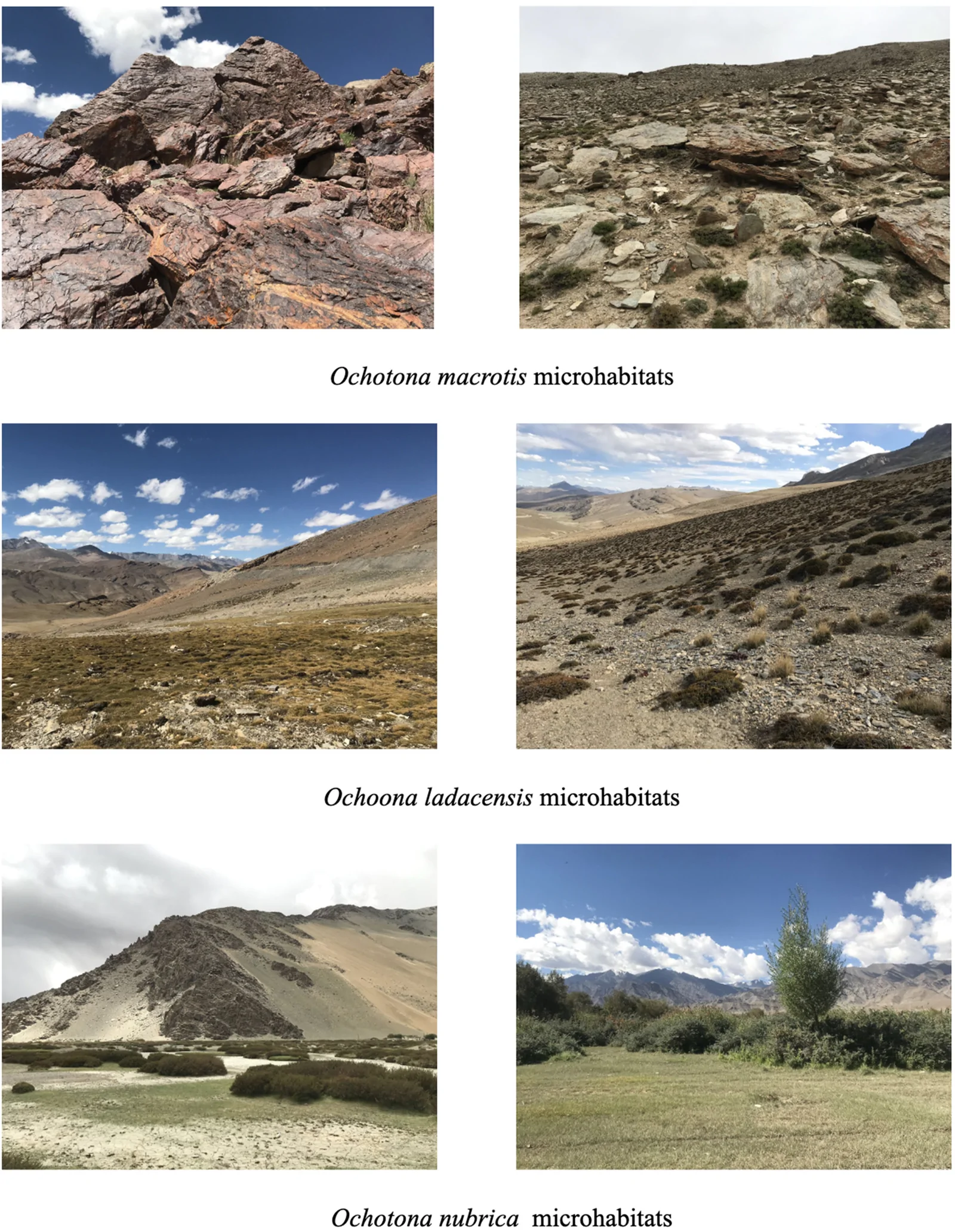
Typical microhabitats of different species of pikas at Ladakh.

— *Ochotona macrotis* (OM): An asocial rock-dwelling pika with a wide elevation range (2300–6000 m). It is known to specialize in rocky talus and scree slopes found in alpine deserts and spruce forests [42].
— *Ochotona nubrica* (ON): a social burrowing species (2800–5300 m). It primarily uses dense, thorny vegetation, including *Hippophae rhamnoides*, *Tamarix* spp.*, Salix* spp.*, Caragana* spp. and *Lonicera* spp., although it utilizes rocks when available [42,43].
— *Ochotona ladacensis* (OL): A social, high-elevation burrowing specialist (4200–5400 m). This species has been described to be unlike the open grassland species *O. koslowi* and is known to burrow under rocks in barren xeric alpine valleys characterized by a complex mosaic of three distinct vegetation communities/microhabitats: (i) barren lands occupied by cushion plants (*Primula* spp.*, Arenaria musciformis, Gentiana* spp.*, Leontopodium* spp.) and sedges (*Carex moorcroftii*); (ii) catmint (*Nepeta* spp.) stands; and (iii) low shrub vegetation consisting of *C*. *versicolor* and *Krascheninnikovia ceratoides* [42,43].

While OM and OL (clade *Conothoa*) diverged around 7 Myr, these species diverged from ON (clade *Ochotona*) around 13 Myr [44,45]. Assuming that closely related species share similar physiological requirements (phylogenetic clustering), OM and OL are expected to use similar bioclimatic and ecological niches [41]. However, the distinct functional differences, observed habitat specialization derived from natural history studies, and our pilot field surveys strongly suggest that local competitive interactions and habitat filtering may override phylogenetic signals. Therefore, this study aimed to quantify how microhabitat partitioning shapes the niches of these three species when examined at different spatial scales.

Based on the functional morphology, evolutionary history, published natural history accounts of the study species and pilot surveys, we formulated the following four testable ecological predictions: These predictions were tested using bioclimatic niche metrics, ecological niche models (topography and vegetation data from paired quadrats), and quantified niche width/overlap metrics across the regional and local spatial scales.

— *Hypothesis 1* (phylogenetic dispersion versus functional divergence). We first tested the influence of evolutionary history on functional ecology. Our prediction was that *O. macrotis* (OM) and *O. ladacensis* (OL) would exhibit dissimilar ecological niches, specifically showing minimal overlap in microhabitat variables (e.g. rock cover vs. vegetation density). We tested whether functional divergence—the established lifestyle difference between the rock-dwelling OM and the burrowing OL—is a stronger ecological filter than their recent phylogenetic relationship (approx. 7 Ma, clade *Conothoa*), which would otherwise predict niche similarity (phylogenetic clustering) [18,19,41].
— *Hypothesis 2* (regional niche width and scale-dependent specialization). We predict that the widely distributed OM and ON will exhibit significantly larger niche breadths than OL at the regional scale (reflecting a known geographic range). Conversely, at the local scale in sympatry, we predict niche contraction (smaller niche size) for species in response to local competitive interactions, resulting in localized specialization.
— *Hypothesis 3*. We predict that a significant overlap in ecological niche space (≥5% niche overlap) will occur between OL and ON at high elevations (≥4200 m). This is hypothesized because both pilot observations and existing literature have indicated that both species utilize the same alpine scrub habitats (particularly Caragana spp.) for cover or resources despite their different refuge construction styles [42].
— *Hypothesis 4*. We predicted that there would be no significant ecological niche overlap (<5% niche overlap) between ON and OM at low elevations (≤3800 m). This prediction is based on the complete functional segregation observed in the microhabitat literature: ON uses dense, tall shrub cover for foraging and refuge, whereas OM uses exposed rocky talus. This distinct partitioning is hypothesized to be a sufficient mechanism to ensure co-existence with minimal niche space overlap [42].

This study not only enhances our understanding of ecological competition and the nuances involved in categorizing species as generalists or specialists but also provides an exploration of proximate factors that drive presence and directly feed back into conservation planning [46,47].

## 2. Methods

### 2.1. Study sites and sampling design

We studied pikas across a span of mountain ranges in the Trans-Himalayas of Ladakh, India, spanning high elevation ranges in the southeast and low elevations in the northwest. For the purpose of our analysis, we define our spatial scales as

— *Regional scale*. This represents the broad realized niche of a species, encompassing all sampled locations across its entire elevational distribution within the study area (e.g. all 2800—6000 m sites for OM).
— *Local scale*. This represents the specialized realized niche, constrained to different elevational zones (high elevation: 4200–6000 m; low elevation: 2800–4200 m). This setup allows us to contrast regional niche breadths with localized specialization due to sympatry-induced competition.

Signs of pika activity (animal sightings, faecal pellets, haypiles and burrows) were documented in the Ladakh landscape between 2017 and 2022 at more than 300 sites. Based on logistic and pilot surveys, a subset of these sites was sampled to document the niche spaces of the three pika species. These sites were chosen to stratify across elevational distribution ranges and zones of sympatry/allopatry (presence plots: 83 plots; random plots: 63 plots) (figures 2 and 3). The use of both presence and random plots provides the true presence/absence data necessary for robust predictive modelling. Although OM largely occurs in allopatry at low elevations, small zones of sympatry with ON occur along the Indus River. At high elevations, different zones of sympatry comprised (a) OM and ON, (b) OM and OL, and (c) OM, ON and OL (figure 2).

**Figure 2.**
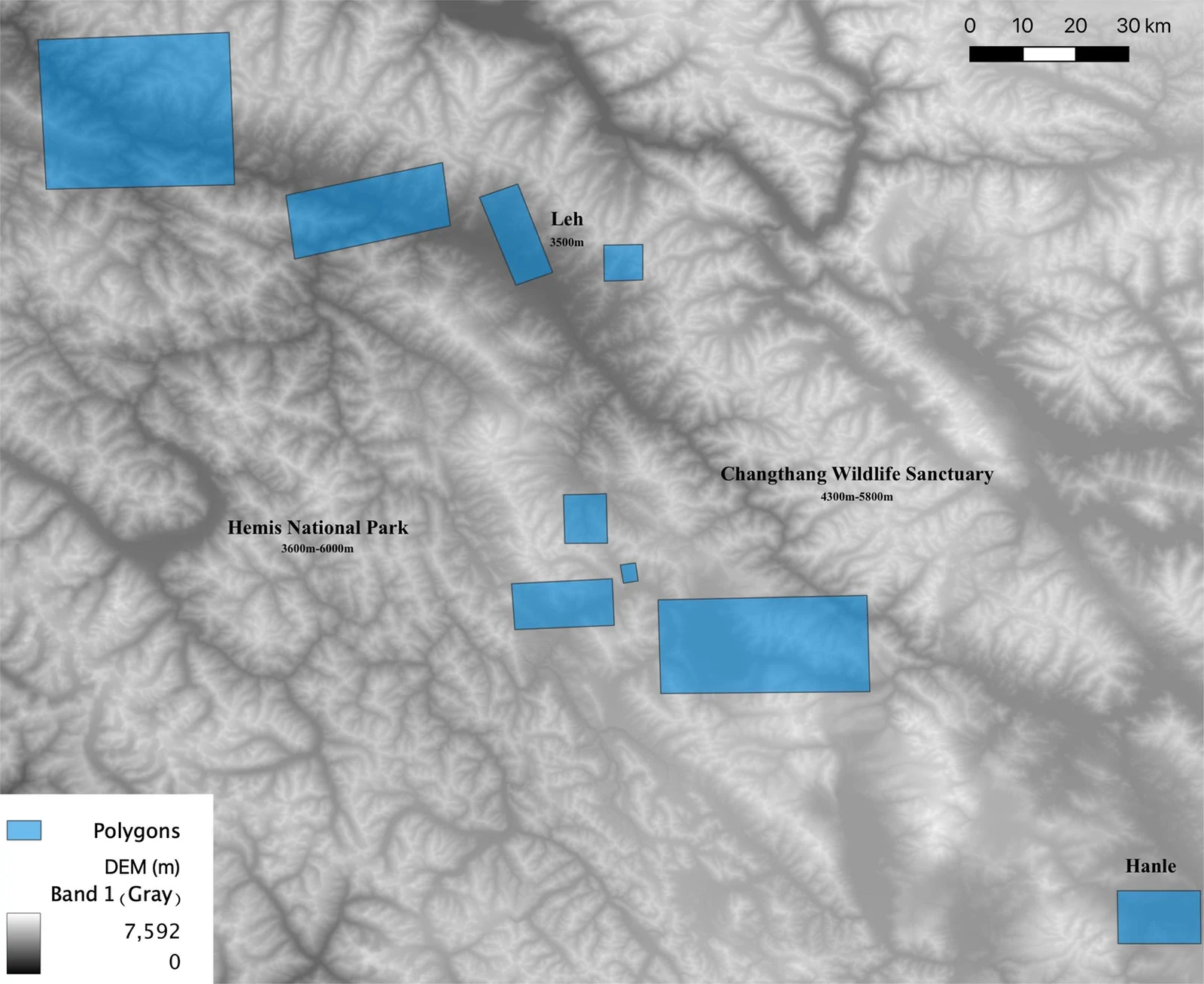
Map of regions sampled in the Trans-Himalayas of Ladakh Union Territory (blue polygons).

**Figure 3.**
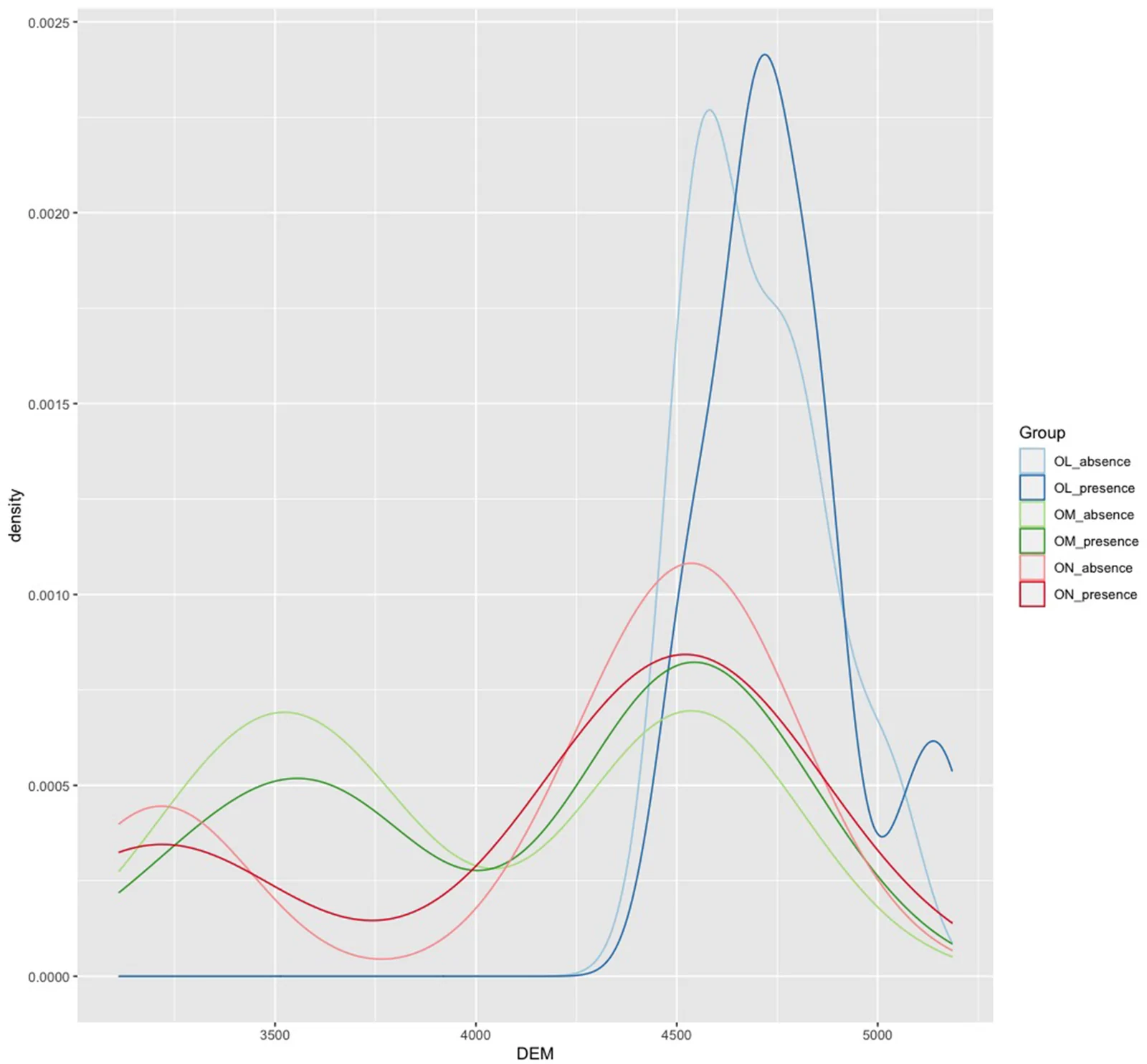
Elevational distribution of presence and absence plots laid in the study across species. OL, *O. ladacensis*; OM, *O.macrotis*; ON, *O. nubrica*.

### 2.2. Data collection and datasets

We collected two primary types of environmental data: broad-scale environmental correlates (used for the bioclimatic niche) and local-scale microhabitat metrics (used for the ecological niche).

#### 2.2.1. Broad-scale environmental correlates (bioclimatic and topographic data)

These data were extracted remotely to define the large-scale climatic envelope and general topographic factors influencing the presence of pikas on a regional scale.

— *Bioclimatic variables*. The CHELSA Bioclim dataset (1981–2010) [48] was downloaded at 30 m resolution, and the pixel values for sampling points were extracted using the Raster Analysis plugin on QGIS (3.34.1-Prizren) (QGIS Development Team [49]) for all 19 variables.
— *Topographic variables*. A digital elevation model (30 m resolution) for our study area (32.5443–34.5757° N, 76.4641–79.3645° E) was downloaded from the Bhuvan, Indian Geo Platform of ISRO (https://bhuvan.nrsc.gov.in/home/index.php). ArcMap 10.5 (Esri [50]) was used to process this raster to obtain the slope and aspect (relevant ecological attributes of a landscape that animals select for residence). These attributes were extracted for all sampled points.
— *Normalized difference vegetation index* (NDVI). NDVI values (30 m resolution) for each sampled point were obtained for the months of July–August (summer) and November–December (winter) (2013–2021) from Landsat 8 imagery (US Geological Survey) with the least cloud cover using Google Earth Engine [51]. These were further processed to provide additional variables concerning the NDVI (supplementary material, table S1).

#### 2.2.2. Local-scale microhabitat metrics (paired quadrat data)

These variables were collected via field surveys using a paired quadrat design at each sampled site to define the precise microhabitat features that determine species' realized niches and partitioning.

In each plot, we quantified physical and vegetative characteristics within a defined quadrat area. To minimize bias and estimate cover accurately, the quadrat area was visually partitioned into four equal sub-quadrats (25% slices). Different plot variables were visually estimated and documented within each sub-quadrat (supplementary material, table S1).

These plot variables include

— *Rock/refuge features*: density, size, and percent cover of rocks (e.g. *perc_rocks*, *number_large_rocks*).
— *Vegetation/resource features*: percent cover and height of different vegetation classes (e.g. *perc_vegetation* and *height_shrub*).
— *Geographic/topographic features*: in-field measurements of elevation and slope.

### 2.3. Statistical analysis and niche modelling

#### 2.3.1. Generalized linear models for presence/absence

We modelled the presence of pikas at sites using logistic regressions with plot variables (supplementary material, Table S1) as predictors using the ‘glm’ function in the R package ‘arm’ [52]. To identify the ecological factors that support the presence of different species of pikas at a broader spatial scale, quadrats sampled across species and sites (*n* = 148) were pooled and scored for a binary presence/absence variable for each species (for instance, presence and absence plots of ON and OL were treated as absence points for OM) (see figure 3). Such an approach is helpful as it is informative regarding where a species is both likely and unlikely to be present.

#### 2.3.2. Spatial extents modelled for generalized linear models

To capture niche spaces of species across elevations while accounting for different sympatric zones, we modelled the presence of species at three different spatial extents: (a) high elevations (local scale), (b) low elevations (local scale) and (c) all Ladakh (regional scale). Because only OM and ON are found at low elevations, only the presence of these species was modelled at this spatial scale. At high elevations, the occurrence of all three species was modelled as they co-occur in this landscape.

#### 2.3.3. Model selection strategy

Owing to problems associated with sample size, all variables could not be subject to a stepwise/backward selection algorithm. To resolve this problem, we used a modified version of the purposeful selection framework [53,54]. The workflow used is as follows:

— Predictor variables that affected presence were identified using univariate logistic regression and plots (one predictor and one binary response variable). All variables that explained the occurrence of a species in these models (*p* < 0.05) were added to the model occurrence (model 1: statistical model). In addition, all variables with a *p*-value cut-off of 0.25 and other variables that were biologically relevant (based on field observations) were additively used to model occurrence (model 2: purposeful model) following [54]. Finally, a third model was constructed with predictors (*p*-value < 0.25 in univariate regressions) binned into different ecological classes (supplementary material, Table S2) to depict the different landscape-level features (model 3: sub-model) that could influence pika presence.
— Stepwise regression (StepAIC) was run on all three models to select the models that best explained the occurrence (based on the three species of interest). When Akaike information criterion (AIC) values varied marginally across the sub-models in the ecological classes, all predictors across the models were considered for further analysis.
— For the sub-model alone, variables from all the best sub-models across different ecological classes were additively used to model presence using stepwise regression for the second time. Care was taken to add/replace biologically relevant variables (based on field experience and literature), even if they were dropped during the sub-modelling process.
— The best models across all modelling approaches were compared and analysed. Finally, biologically relevant interactions (based on field observations) were evaluated based on the AIC scores. Goodness-of-fit tests [55] were performed to assess the model fit. Standardized regression coefficients were examined to determine the relative importance of the different variables in the best models.

#### 2.3.4. Niche overlap and width quantification (Bayesian approach)

*Bioclimatic niche*. A dataset was constructed by filtering out all absence records and retaining only the presence records. Bioclimatic variables extracted for these presence points were used to construct a climatic niche after removing the correlated variables. These uncorrelated bioclimatic variables were subjected to a principal component analysis (PCA) for data dimensionality reduction, with the first three axes retained for further analysis (explaining more than 80% of the total variance) of niche space, overlap and widths.

*Ecological niche.* While constructing the ecological niche, only variables that were both biologically (based on natural history observations) and statistically important (based on univariate regressions for each species) for the presence of each species (OM, variables related to rocks and geography; ON, variables related to vegetation and shrubs in particular; OL, variables related to vegetation and elevation) (variables highlighted in blue in supplementary material, table S2) were retained. This dataset was subjected to PCA to reduce dimensionality and visualize the variation along eigenvectors. Variables related to vegetation (perc_vegetation, height_tallgrass_reeds, height_shrub and average NDVI in summer) and rocks (perc_rocks) loaded onto PC1, while PC2 and PC3 were driven by only one variable each, elevation and variance in winter NDVI, respectively (cos2 > 0.5). Because the three principal component axes explained 55% of the total variance in the dataset, only three dimensions of the principal component transformed points were considered for further analysis of niche overlap and niche width. PCA revealed the separation of broad niches along different axes of rocks, vegetation, elevation and NDVI.

*Bayesian inference of niche space, overlaps, and width.* We used the NicheRover package in R [56] with a Bayesian-based probabilistic estimation of niche space, overlaps and width [57]. A specified number of Monte Carlo computed directional overlaps were drawn from the parameter list (α = 0.95), indicating the probability of finding ‘species A’ in the niche space of ‘species B’. The niche width was obtained by calculating the posterior distribution of niche size by species (niche size for every posterior sample drawn) [57]. Niche estimation following the methods described was performed at two scales (regional scale: all of Ladakh, three species representing three niches) and (local scales: ON and OM—one niche at high elevation and one niche at low elevation; OL— one niche at high elevation—a total of five niches).

## 3. Results

### 3.1. Distribution and sympatry of pikas

While at low-mid elevations (3000—4200 m) in the northwest, OM was found largely in allopatry; both OM and ON occurred at moderate proximity (approx. 1 km) along the Indus River in central Ladakh. At high elevations (>4200 m) in the southeast, different pairs of species were found in very close proximity, forming complex zones of sympatry with two or three species in different combinations: (a) ON and OM, (b) OL and OM, and (c) ON, OM and OL (approx. 200 m) (figure 2).

### 3.2. Occurrence modelling: ecological drivers of presence

We used generalized linear models (GLMs) to identify the ecological factors supporting species presence across three spatial scales: regional (all Ladakh), local high elevation and local low elevation. The purposeful modelling approach (model 2) consistently provided the best-fitting models (lowest AIC) across most spatial scales for all three species (tables 1–3).

**Table 1.** Best multivariate models found using the StepAIC function across different modelling methods (see models 1–3 in methods) and spatial scales for *O. macrotis*. Within each spatial level, the best model and the approach are highlighted.

modelling framework	best model	AIC	top contributors to model
spatial level: regional			
statistical model	macrotis ∼ perc_rocks + num_4rocks + DEM.elevation + x̄ (NDVI_Winter)	94.25	
purposeful model	macrotis ∼ perc_2rocks + perc_4rocks + num_4rocks + perc_herb_forb + perc_shrub + height_tallgrass_reeds + x̄ (NDVI_Winter) + x̄ (NDVI_Summer) + stdev (NDVI_Summer)	89.41	perc_4rocks num_4rocks x̄ (NDVI_Summer)
sub-model	macrotis ∼ perc_2rocks + perc_4rocks + num_4rocks + perc_herb_forb + perc_shrub + perc_tallgrass_reeds + height_tallgrass_reeds + x̄ (NDVI_Winter) + x̄ (NDVI_Summer) + stdev (NDVI_Summer)	88.5	perc_4rocks num_4rocks x̄ (NDVI_Summer)
spatial level: Local_ high elevations			
statistical model	macrotis ∼ perc_3rocks + perc_4rocks + DEM.elevation	29.74	
purposeful model	macrotis ∼ perc_rocks + perc_3rocks + perc_4rocks + num_4rocks + perc_vegetation + perc_shrub + stdev (NDVI_Winter)+ DEM.elevation	18	Perc_4rocks num_4rocks DEM.elevation Perc_shrub stdev (NDVI_Winter)
sub-model	macrotis ∼ perc_3rocks + perc_4rocks + DEM.elevation	29.74	
spatial level: Local_ low elevations			
statistical model	macrotis ∼ num_4rocks + perc_4rocks + x̄ (NDVI_Winter)	45.94	
purposeful model	macrotis ∼ perc_bareground + perc_4rocks + num_3rocks + num_4rocks + perc_vegetation + perc_shrub + perc_herb_forb + perc_tallgrass_reeds + height_shrub + height_herb_forb + height_tallgrass_reeds + num_tallgrass_reeds + num_shortgrass_sedge + num_3rocks + perc_bareground + x̄ (NDVI_Winter) + x̄ (NDVI_Summer) + stdev (NDVI_Summer) + x̄ (Δ NDVI) + DEM.elevation	38	all variables are equally important
sub-model	macrotis ∼ num_4rocks + perc_4rocks + perc_tallgrass_reeds + num_shortgrass_sedge Average.Delta.NDVI	45.5

**Table 2.** Best multivariate models found using the StepAIC function across different modelling methods (see models 1–3 in Methods) and spatial scales for *O. nubrica*. Within each spatial level, the best model and the approach are highlighted.

modelling framework	best model	AIC	top contributors to model
spatial level: regional			
statistical model	nubrica ∼ perc_vegetation + perc_rocks + num_3rocks + num_4rocks + num_shortgrass_sedge + perc_herb_forb + perc_shrub + height_tallgrass_reeds + height_shrub + height_trees + Slope + x̄ (NDVI_Winter)	26	
purposeful model	nubrica ∼ perc_vegetation + perc_3rocks + perc_4rocks + perc_herb_forb + perc_shrub + height_shrub + x̄ (NDVI_Winter) + x̄ (NDVI_Summer)+ x̄ (Δ NDVI)	20	all variables are equally important besides variables related to rocks.
sub-model	nubrica ∼ perc_3rocks + perc_4rocks + perc_herb_forb + perc_shrub + x̄ (NDVI_Summer) + num_shrub + height_shortgrass_sedge + height_tallgrass_reeds + height_shrub	20	all variables are equally important besides x̄ (NDVI_Summer)
spatial level: Local_high elevations			
statistical model	nubrica ∼ num_shrub + perc_herb_forb + perc_shrub + height_tallgrass_reeds + height_shrub + Average.NDVI_Nov_Dec	20.12	
purposeful model	nubrica ∼ perc_bareground + perc_3rocks + perc_shrub + height_herb_forb + height_shrub + Average.NDVI_Nov_Dec	14	
sub-model	nubrica ∼ perc_vegetation + perc_shrub + x̄ (NDVI_Winter)+ height_shrub	10	all variables are equally important
Spatial level: Local_ low elevations			
statistical model	nubrica ∼ height_tallgrass_reeds + stdev (NDVI)	6	all variables are equally important
purposeful model	nubrica ∼ height_tallgrass_reeds + x̄ (Δ NDVI)	6	all variables are equally important
sub-model	nubrica ∼ x̄ (NDVI_Summer) + height_tallgrass_reeds	6	all variables are equally important

**Table 3.** Best multivariate models found using the StepAIC function across different modelling methods (see models 1–3 in Methods) and spatial scales for *O. ladacensis*. Within each spatial level, the best model and the approach are highlighted.

modelling framework	best model	AIC	top contributors to model
spatial level: regional			
statistical model	ladacensis ∼ num_4rocks + DEM.elevation	80.95	
purposeful model	ladacensis ∼ perc_bareground + perc_vegetation + perc_rocks + perc_1rocks + perc_4rocks + num_4rocks + perc_trees + height_shortgrass_sedge + DEM.elevation + Aspect + x̄ (NDVI_Winter)	63.99	all variables equally important
sub-model	ladacensis ∼ perc_1rocks + num_4rocks + height_shrub + DEM.elevation + Aspect	73.37	
spatial level: Local_ high elevations			
statistical model	ladacensis ∼ DEM.elevation + Slope	89.94	
purposeful model	ladacensis ∼ perc_1rocks + perc_4rocks + height_shrub + DEM.elevation + Slope	71.93	perc_1rocks perc_4rocks DEM.elevation Slope
sub-model	ladacensis ∼ perc_rocks + perc_1rocks + perc_4rocks + perc_cushionplant + height_shrub	79.5	

#### 3.2.1. *Ochotona macrotis*: the rock specialist

The presence of OM was consistently predicted by rock-related features across all spatial scales examined, reinforcing its role as an obligate rock-dwelling specialist.

*Univariate models*. A comparison of univariate models at different spatial scales (supplementary material, table S3) revealed that a low percentage cover of vegetation, a greater percentage cover of rocks with large rocks in particular and a greater number of large-sized rocks were important for predicting the presence of OM across all spatial scales. At a larger regional scale, the presence of this species was positively associated with slope. At high elevations, the presence was positively associated with the coverage of moderately sized rocks (class 2). At low elevations, presence was negatively associated with NDVI (supplementary material, table S3).

*Multivariate models.* At a larger regional scale, the presence of OM was additionally supported by a larger cover of large rocks and lower NDVI in summer; at high elevations by a higher cover of large rocks, a lower cover of shrubs and consistently low NDVI in winter; and at low elevations, the occurrence was driven by many variables (table 1).

#### 3.2.2. Ochotona nubrica

The presence of ON was consistently driven by tall vegetation and dense shrub cover across all spatial scales, making it an obligatory shrub specialist.

*Univariate models*. A comparison of univariate models (supplementary material, table S4) showed that a large percentage of bush cover, a large number of shrubs, taller shrubs, and tall grass/reeds were associated with the presence of ON across all scales. The presence at the larger regional scale was associated with rock cover (negatively), large-sized rocks (negatively) and higher NDVI across seasons (positively)*.* At low elevations, the presence was positively associated with average NDVI (positively) and reflected greener habitats occupied by the species. At high elevations, tall shrubs and a large number of shrubs are good predictors of presence.

*Multivariate models*. At a larger regional scale, variables that predicted both the absence (rocks) and the presence of the species (shrubs and NDVI) were important (table 2). At high elevations, only shrub height (positive) and shrub cover (positive) were sufficient to explain the presence of the species (table 2). At low elevations, NDVI and vegetation (tall grass and reeds) were necessary to explain presence, although the species was restricted to large stands of sea buckthorn (figure 1, bottom right; table 2).

#### 3.2.3. Ochotona ladacensis

The occurrence of OL was strongly linked to topographic variables of elevation and slope and was characterized by a consistent avoidance of large rock features at both regional and local scales.

*Univariate models*. At both regional and local scales, the presence of OL was associated with higher elevations (supplementary material, Table S5). At a larger regional scale, a lower percentage cover of rocks, a lower percentage number and cover of large-sized rocks, short herb-forbs and tall cushion plants were associated with the presence of the species. At high elevations, only topographic variables, such as elevation (positive) and slope (positive), predicted presence (supplementary material, table S5).

*Multivariate models*. At both the regional and local scales, variables that predicted the absence and presence of the species were equally important. At both scales, the presence of OL was additively associated with ecological factors identified in the univariate models (less cover of rocks, smaller-sized rocks, short-grazed vegetation, tall cushion plants, higher slope and elevation).

### 3.3. Comparison of climatic/ecological niche overlaps and widths between species at different spatial scales

#### 3.3.1. Bioclimatic niches (CHELSA climatology)

Projections of bioclimatic niche space at regional scales showed no discernible separation between species and were characterized by large degrees of overlap (figure 4*a*). There was a high probability associated with finding both OL and ON in the niche of OM (mean > 60%), an intermediate probability associated with finding OL and OM in the niche of ON (mean approx. 40%), and a lower probability associated with finding OM and ON in the bioclimatic niche of OL (mean approx. 30%) (figure 4*b*). The niche width of OM was the largest, followed by ON and finally OL, mirroring the distribution ranges of species within Ladakh (figure 4*c*).

**Figure 4.**
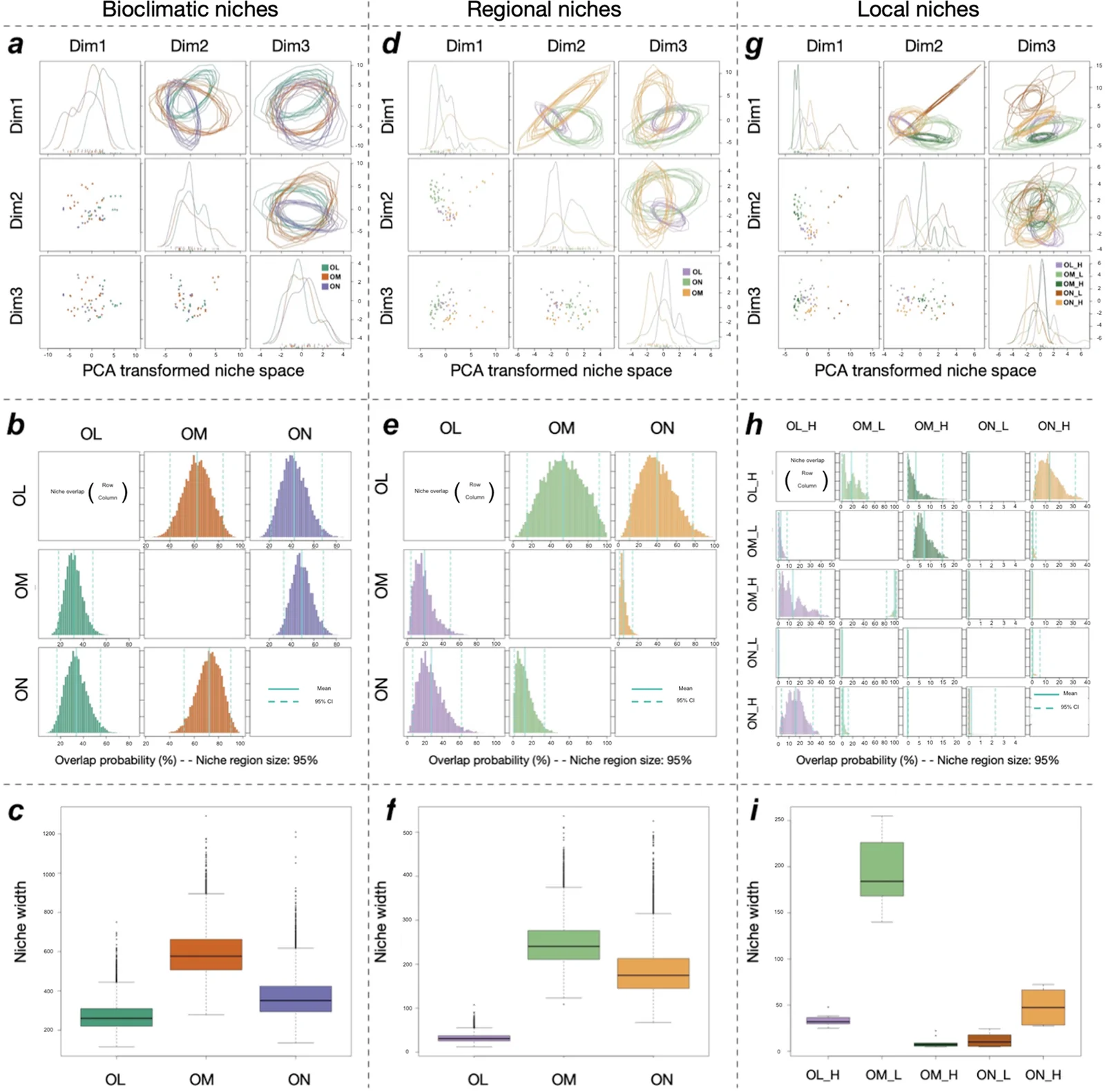
(*a*,*d*,*g*) Ten elliptical projections of niche space constructed using principal component transformed axes for each species/population pair (NicheRover). (*b*,*e*,*h*) Directional niche overlaps between species/ population pairs (probability of species 1 being found in the niche of species 2). (*c*,*f*,*i*) Niche width for each species/population (*n*-dimensional hypervolume). Panels show (*a*–*c*) bioclimatic niches at regional scales, (*d*–*f*) ecological niches at regional scales and (*g*–*i*) ecological niches at local scales. Legend: OL, *O. ladacensis*; ON, *O. nubrica*; OM, *O. macrotis*; OL-H, *O. ladacensis* high elevation; ON-H, *O. nubrica* high elevation; ON-L, *O. nubrica* low elevation; OM-H, *O. macrotis* high elevation; OM-L, *O. macrotis* low elevation.

#### 3.3.2. Ecological niches (topographic and vegetation features)

*Regional scale*. Elliptical projections of niche space indicated that the three species separated best along the first two dimensions, albeit with significant overlap (figure 4*d*). OL was very likely to be found in the microhabitats that were inhabited by ON (mean = 40%) and OM (mean = 50%)*,* with both OM and ON being less likely to be found in microhabitats inhabited by other species (mean < 15%) (figure 4*e*). The niche width was found to be different across species (Pr(>*F*) = <2e−16 ***, *F* = 6541, df = 2), with OL having the smallest niche, ON having an intermediate-sized niche, and OM having the largest niche, as evidenced by Tukey's HSD tests (figure 4*f*).

*Local scale (high elevation–low elevation)*. Populations of species were subdivided by elevation (OL: high elevations alone; ON and OM: low and high elevations, respectively). Elliptical projection of niche spaces highlighted overlaps/similarities between the niches of (a) ON and OL at high elevations and (b) OM at low and high elevations (figure 4*g*). On the other hand, ON at low elevations had the most distinct niche and little to no overlap with other species across elevations (figure 4*g*). Species at local scales were unlikely to be found in microhabitats of other species (overlap probability < 20%), and the largest overlaps in niches were at high elevations (overlap probability: 5–15%) (figure 4*h*). The niche widths of species at the local scale were significantly different (Pr(>*F*) = <2e−16 ***, *F* = 9570, df = 4), and revealed contrasting patterns to those recovered at the broad scale. OM at low elevations was inferred to have the largest niche size (largely in allopatry), followed by ON and OL at high elevations, with the second- and third-largest niches, respectively (figure 4*i*). The niche width of ON at low elevations and OM at high elevations was similar and was among the smallest niches in comparison (figure 4*i*).

## 4. Discussion

The study set out to examine realized Hutchinsonian niches of different species of pikas across regional (large) and local (small) spatial scales, providing a quantitative framework for understanding how they navigate ecological competition in the Trans-Himalayas. We documented microhabitat–species associations and described niche spaces and directional overlaps at different spatial scales using a paired quadrat design. While some species at regional scales were inferred to be generalists, populations of the same species were inferred to be specialists at smaller spatial scales. Over and above metrics, such as niche widths and overlaps to place species on the generalist–specialist continuum, the spatial scale at which the problem is examined is also important. We recovered broad patterns of microhabitat partitioning at local scales, with a closer look at asymmetric competition between species pairs (changes in niche use and directional overlaps). This is one of the few studies that explores scale-dependent phenomena while describing niche spaces and overlaps between species [16,17].

### 4.1. Validation of model selection

Across modelling methods used to describe the presence of species using a GLM framework, the purposeful modelling approach (which takes into account both statistically and ecologically relevant variables) consistently produced models with the lowest AIC (tables 1–3) [53,54]. This consistency validates the ecological relevance of the factors identified in the best-fit model.

#### 4.1.1. Functional divergence overrides phylogenetic signal (hypothesis 1)

At a broad regional scale, all three species exhibited large, highly overlapping bioclimatic niches (figure 4*a*,*b*). This indicates that the climatic space available to pikas is broadly shared, given the large geographic distribution of these species. However, our ecological niche analysis found evidence for the divergence of niches between the closely related OL and OM. This functional divergence is driven by distinct microhabitat requirements, which act as the primary filtering mechanisms separating the realized niches of these closely related species.

OM's presence across all scales was robustly predicted by the cover and number of large rocks (table 1). Crucially, the top predictive models consistently favoured rock features (refuges) over resources. This strong reliance on rock refuges is consistent with other studies on *O. princeps* and *O. roylei* where rocks are chosen for their thermal buffering capabilities and escape from predators [58–60]. Site selection by *O. princeps* is strongly dependent on the insulating properties of rock refuges that buffer larger degrees of ambient temperatures, which provides a functional explanation for the dominance of rock features in our models [60].

In sharp contrast to OM, the closely related OL has diverged functionally and occupies a burrowing niche associated with xeric alpine valleys, alpine scrubs and alpine meadows/grasslands at high slopes and elevations (table 3). This species was found to construct burrows in different microhabitats, such as (a) under rocks in xeric alpine valleys and mountains, (b) at the base of stunted *Caragana* spp. bushes and (c) in open alpine grasslands/meadows similar to those built by *O*. *koslowi* (use of this habitat has not been recorded previously) [42]. This functional divergence is the dominant factor separating the realized niches, overriding the shared evolutionary history.

#### 4.1.2. Scale-dependent specialization and niche shifts in response to competition (hypothesis 2)

Niche width analysis revealed that the categorization of species along the generalist–specialist continuum is dependent on the spatial scale of examination, supporting the idea that local competitive interactions shrink niche space [16,17].

Regional generalists are local specialists. At a larger regional scale, the niche widths of OM and ON were larger than those of OL, accurately describing the global geographical distributions of these species. At the local scale, the niches of ON and OL were larger than those of OM at high elevations.

At low elevations, where OM largely occurred in allopatry, it had the largest niche width (generalist). This generalist niche was supported by several vegetation- and rock-related variables in the predictive models (table 1). Conversely, where it occurred in multispecies sympatry (high elevations), it had the smallest niche width (specialist) (figure 4*i*). This specialization was characterized by low cover of shrubs and lower variation in winter NDVI in the best-fit models (table 1). This significant reduction in niche size is the classic ecological signature of competitive compression, indicating that local interactions force OM into a narrower, more specialized rock refuge niche when competitors are present [21–23].

ON exhibits a large niche width regionally and is strongly associated with tall stands of alpine shrub habitats at both low and high elevations [42]. At high elevations, this niche was characterized by large vegetation cover (thorny shrubs in particular), tall stands of shrubs and arid landscapes with low winter NDVI; at low elevations, it was strongly associated with tall stands of sea buckthorn scrub associated with high winter NDVI (*Hippophae rhamnoides*), depicting landscape-level differences in vegetation and moisture across elevations (table 2). The OL exhibited the smallest niche width at the regional scale because it was an elevational specialist (>4200 m). However, its intermediate niche width at the local scale suggests that it is a microhabitat generalist within its high-elevation zone, successfully utilizing many microhabitats such as open steppe grasslands, Xeric alpine valleys with rocks, and *Caragana* scrub, a nuance that has never been seen in this light.

#### 4.1.3. Microhabitat partitioning and asymmetric competition (hypotheses 3 and 4)

The low niche overlaps observed at the local scale (<20%, figure 4*h*) confirm strong microhabitat segregation across communities, highlighting the importance of partitioning for co-existence.

At high elevations, ON and OL showed the largest niche overlaps (5–15%), given that they share the Caragana alpine scrub habitat, as expected (hypothesis 3; figure 4*h*). We documented strong niche segregation at low elevations (hypothesis 4) where OM and ON co-occurred. They occupied highly distinct niches with little to no overlap (hypothesis 4; figure 4*g*,*h*) and largely differed in their use of refuges. While OM strictly used rock crevices at both high and low elevations, ON at low elevations constructed forms (shallow scrapes in soil similar to those used by hares), and those at high elevations constructed both burrow systems and used forms. At the regional scale, OL was highly likely to be found in the microhabitats of OM and ON (40–50% probability), but OM and ON were less likely to be found in OL microhabitats (<15%). This asymmetry suggests that OL, an elevational specialist, is either a superior competitor or the most adaptable species, capable of utilizing resources to which OM and ON are less restricted, a pattern often seen in competition (figure 4*e*).

The observed segregation is likely reinforced by predation risk. Given the high diversity of observed predators, including the red fox (*Vulpes vulpes*), wolf (*Canis cf. lupus*), mountain weasel (*Mustela altaica*), little owl (*Athene noctua*), Saker falcon (*Falco cherrug*) and upland buzzard (*Buteo hemilasius*) (observed between 2017 and 2023 by our team), species-specific microhabitat choices (large rocks for OM, dense shrubs for ON) likely represent an optimized predation risk-resource trade-off [61,62], where these features provide reliable cover from predation pressure.

This study provides the first quantitative analysis of realized niche spaces and overlaps among three sympatric species of pika from Asia. This illustrates that sympatric pika communities in the Tibetan marginal mountains of Ladakh, India segregate along rocks, vegetation and elevation. The core finding—that species shift from being regional generalists to local specialists in response to co-occurrence—highlights the power of spatially explicit considerations in identifying potential asymmetric competition that has structured realized niche spaces of species. A nuanced understanding of species–microhabitat associations and factors that promote sympatry is likely to be very useful for the conservation of cold-adapted pika specialists in the rapidly developing Trans-Himalayan landscape.

## Supplementary Material



## Data Availability

Data and relevant code for this research work are stored in GitHub [63] and have been archived within the Zenodo repository [64]. Supplementary material is available online [65].
