# Supplementary material for: Sympatric pikas partition microhabitats differently across a spatial-elevational gradient
Source: R Soc Open Sci. 2026 Oct 14;13(10):250865. doi: 10.1098/rsos.250865 (PMC13682429; doi:10.1098/rsos.250865)
Supplement: Supplementary file 1 [file rsos.250865.zip › rsos.250865/rsos250865_si_001.docx]

**Supplementary material**

Manuscript Title: Sympatric pikas partition microhabitats differently across a spatial-elevational gradient

Table S1: Plot features and variables measured in each 10m*10m plot. Rocks were sub-categorized based on standard sizes. Vegetation was further sub-categorized into short grasses/sedges, tall grasses/reeds, herbs/forbs and shrubs. Rocks were categorically classified based on size using a standard construction helmet (Size 1 in red color) depicted in the image below.

| **Plot features** | **Variables documented** | | | | |
| --- | --- | --- | --- | --- | --- |
| **Rocks**  Class 1  Class 2  Class 3  Class 4 or larger | Total % rock cover | % cover by rocks of different sizes | Number of large-sized rocks |  |  |
| **Vegetation**  Short grass/Sedges  Tall grass/Reeds  Herbs/Forbs  Shrubs  Cushion plants  Trees | Number of plants in each category of vegetation | Maximum height of plants in each category of vegetation | x̄ (Δ NDVI)  stdev (NDVI)  x̄ (NDVI_Winter)  stdev (NDVI_Winter)  x̄ (NDVI_Summer))  stdev (NDVI_Summer) | Total % bareground cover  Total % vegetation cover | % cover of each category of vegetation |
| **Topographic features** | Elevation (m) | Slope | Aspect |  |  |

Table S2: Model predictors in different ecological classes that depict different landscape features that could potentially influence occupancy by the different species of pika. Biologically and statistically important variables used to construct niche space, estimate overlaps, and widths have been italicized.

| **Ecological classes** | **Variables** |
| --- | --- |
| **Rocks** | % total rock cover  % Class 1 rock cover  % Class 2 rock cover  *% Class 3 rock cover*  *% Class 4 rock cover*  *Num_class3rocks*  *Num_class4rocks* |
| **Geographic features** | *Slope*  Aspect  *Elevation (DEM)*  Distance to water |
| **Vegetation_1** | Num_shortgrass_sedge  Num_tallgrass_reeds  Num_herb_forb  *Num_shrub*  Num_cushionplant  Num_trees |
| **Vegetation_2** | *% total vegetation cover*  % total bareground  *% shortgrass_sedge*  *% tallgrass_reeds*  *% herb_forb*  *% shrub*  *% cushionplant*  % trees  x̄ (Δ NDVI)  stdev (NDVI)  *x̄ (NDVI_Winter)*  stdev (NDVI_Winter)  *x̄ (NDVI_Summer)*  stdev (NDVI_Summer) |
| **Vegetation_3** | Height _shortgrass_sedge  *Height _tallgrass_reeds*  *Height _herb_forb*  *Height _shrub*  Height _cushionplant  Height _trees |

Table S3: Comparison of variables that significantly (p < 0.05) predicted the occurrence of *Ochotona macrotis* at different spatial scales in univariate logistic regressions. The sign indicates the directionality of the relationship with occurrence.

| **Spatial level** | **Significant variables** | **Variables unique to a spatial scale** | **Common variables across spatial scales** |
| --- | --- | --- | --- |
| **Regional scale**  **Ladakh** | perc_bareground (-)  perc_vegetation (-)  perc_rocks (+)  perc_3rocks (+)  perc_4rocks (+)  num_3rocks (+)  num_4rocks (+)  perc_herb_forb (+)  perc_shrub (-)  DEM elevation (-)  Slope (+)  x̄ (NDVI_Winter) (-) | Slope (+) | perc_vegetation (-)  perc_rocks (+)  perc_4rocks (+)  num_4rocks (+) |
| **Local scale**  **High elevation** | perc_bareground (-)  perc_vegetation (-)  perc_rocks (+)  perc_2rocks (+)  perc_3rocks (+)  perc_4rocks (+)  num_3rocks (+)  num_4rocks (+)  perc_herb_forb (+)  DEM elevation (-) | perc_2rocks (+) |  |
| **Local scale**  **Low elevation** | perc_vegetation (-)  perc_rocks (+)  perc_4rocks (+)  num_4rocks (+)  x̄ (NDVI_Winter) (-)  x̄ (NDVI_Summer) (-)  stdev (NDVI_Summer) (-) | x̄ (NDVI_Summer) (-)  stdev (NDVI_Summer) (-) |  |

Table S4: Comparison of variables that significantly (p < 0.05) predicted the occurrence of *Ochotona nubrica* at different spatial scales in univariate logistic regressions. The sign indicates the directionality of the relationship with occurrence.

| **Spatial level** | **Significant variables** | **Variables unique to a spatial scale** | **Common variables across spatial scales** |
| --- | --- | --- | --- |
| **Regional scale**  **Ladakh** | perc_vegetation (+)  perc_rocks (-)  perc_3rocks (-)  perc_4rocks (-)  num_3rocks (-)  num_4rocks (-)  num_shrub (+)  perc_shortgrass_sedge (-)  perc_herb_forb (-)  perc_shrub (+)  height_tallgrass_reeds (+)  height_herb_forb (+)  height_shrub (+)  height_trees (+)  Slope (-)  x̄ (NDVI_Winter) (+)  x̄ (NDVI_Summer) (+)  stdev (NDVI_Summer) (+) | perc_rocks (-)  perc_3rocks (-)  perc_4rocks (-)  num_3rocks (-)  num_4rocks (-) | num _shrub (+)  perc_shrub (+)  height_shrub (+)  height_tallgrass_reed (+) |
| **Local scale**  **High elevation** | num_shrub (+)  perc_shortgrass_sedge (-)  perc_herb_forb (-)  perc_shrub (+)  height_tallgrass_reeds (+)  height_shrub (+)  x̄ (NDVI_Winter) (+) | None |  |
| **Local scale**  **Low elevation** | perc_vegetation (+)  num_shrub (+)  perc_shrub (+)  height_tallgrass_reeds (+)  height_herb_forb (+)  height_shrub (+)  height_trees (+)  x̄ (Δ NDVI) (+)  stdev (NDVI) (+)  x̄ (NDVI_Winter) (+)  x̄ (NDVI_Summer) (+)  stdev (NDVI_Summer) (+) | x̄ (Δ NDVI) (+)  stdev (NDVI) (+) |  |

Table S5: Comparison of variables that significantly (p < 0.05) predicted the occurrence of *Ochotona ladacensis* at different spatial scales in univariate logistic regressions. The sign indicates the directionality of the relationship with occurrence.

| **Spatial level** | **Significant variables** | **Variables unique to a spatial scale** | **Common variables across spatial scales** |
| --- | --- | --- | --- |
| **Regional scale**  **Ladakh** | perc_rocks (-)  perc_4rocks (-)  num_4rocks (-)  height_herb_forb (-)  height_cushionplant (+)  DEM elevation (+) | perc_rocks (-)  perc_4rocks (-)  num_4rocks (-)  height_herb_forb (-)  height_cushionplant (+) | DEM elevation (+) |
| **Local scale**  **High elevation** | DEM elevation (+)  Slope (+) | Slope (+) |  |
